# 非水毛细管电泳法同时测定消毒剂、个人护理品及药膏中三氯生、三氯卡班和对氯间二甲苯酚

**DOI:** 10.3724/SP.J.1123.2022.06016

**Published:** 2023-02-08

**Authors:** Ruoke JIANG, Xiaojing DING

**Affiliations:** 1.首都医科大学公共卫生学院，北京 100069; 1. School of Public Health，Capital Medical University，Beijing 100069，China; 2.北京市疾病预防控制中心，北京市预防医学研究中心，北京 100013; 2. Beijing Center for Disease Control and Prevention，Beijing Research Center for Preventive Medicine，Beijing 100013，China

**Keywords:** 非水毛细管电泳, 三氯生, 三氯卡班, 对氯间二甲苯酚, 消毒产品, 个人护理品, nonaqueous capillary electrophoresis （NACE）, triclosan （TCS）, triclocarban （TCC）, *p*-chloro-*m*-xylenol （PCMX）, disinfection products, personal care products

## Abstract

三氯生（TCS）、三氯卡班（TCC）和对氯间二甲苯酚（PCMX）这3种消毒有效成分因广谱杀菌而广泛应用于消毒剂、个人护理品及药品中。为监督这些产品质量、保障消毒效果，非常有必要建立同时分析这3种消毒有效成分的方法。鉴于此，该文以十二烷基三甲基溴化铵（DTAB）为电渗流反转剂，建立了同时测定上述3种消毒有效成分的非水毛细管电泳-紫外检测新方法，并对分离缓冲体系及浓度、样品介质等条件进行了优化。结果表明，未涂层熔融石英毛细管（20.0 cm×50.0 μm，总长度30.2 cm）为分离毛细管，14 mmol/L硼砂、2 g/L聚乙二醇20000和0.5 mmol/L DTAB的甲醇溶液为分离缓冲溶液，样品介质为含5 g/L聚乙二醇20000的甲醇-乙腈（50∶50， v/v），在214 nm下进行检测，实现了3种消毒有效成分的同时分离。在1~100 mg/L范围内，3种消毒有效成分的校正峰面积与质量浓度呈良好的线性关系，相关系数（*r*）均大于0.99。方法的检出限（LOD，信噪比为3）为0.2 mg/L，定量限（LOQ，信噪比为10）为1.0 mg/L；加标回收率在94.5%~104.4%范围内，相对标准偏差均小于4.8%。利用研究建立的新方法测定了消毒液、洗手液、婴儿爽身粉、抑菌乳膏共31件样品，并将结果与国家标准方法GB/T 27947-2020和GB/T 34856-2017中的液相色谱法结果相比较，无统计学显著性差异。与国标方法相比，方法极大地减少了有机溶剂消耗量。该法前处理简单，结果准确，非常适合实验室的常规分析。

## Abstract (en)

Triclosan （TCS）， triclocarban （TCC）， and *p*-chloro-*m*-xylenol （PCMX） are some of the most widely used antibiotics because of their broad-spectrum and highly-efficient bactericidal effects. In the context of disinfection， the National Standard GB 38598-2020 stipulates that the contents of the effective ingredients present in a disinfectant must be specified， wherein their range must fall within 90%-110% of the specified central value. To ensure a suitable product quality， analysis by high performance liquid chromatography （HPLC） is recommended by both the GB/T 27947-2020 and GB/T 34856-2017. However， the results analyzed according to the National Standard method often exceed the declared contents， thereby indicating the necessity to establish a new method based on a completely different principle （e. g.， capillary electrophoresis）， especially since it was not possible to analyze TCS， TCC， and PCMX in a single injection using the National Standard method. Moreover， using this method， large amounts of methanol were consumed， which could be potentially harmful to both operators and the environment. In terms of their water solubilities， this decreases in the order of PCMX>TCS>TCC， wherein TCC is insoluble in water. As such， the use of nonaqueous capillary electrophoresis （NACE） based on running buffer solutions prepared in pure organic solvents （e. g.， methanol or acetonitrile） is necessary. In this paper， a new NACE approach combined with an ultraviolet detection method was developed for the simultaneous analysis of TCS， TCC， and PCMX in disinfectants， personal care products， and ointments. For this purpose， an uncoated fused silica capillary （20 cm×50 μm， total length=30.2 cm） was used as the separation column with a separation buffer composed of 14 mmol/L sodium borate， 2 g/L polyethylene glycol （PEG） 20000， and 0.5 mmol/L dodecyltrimethylammonium bromide （DTAB） in methanol. Following optimization of the separation parameters， the complete and simultaneous separation of TCS， TCC， and PCMX was achieved when the sample solution was prepared using 5 g/L PEG 20000 in methanol-acetonitrile （50∶50， v/v）. It was possible to directly inject the sample into the analysis system after a simple dilution with the sample medium， and no interference was observed in any of the sample electropherograms when a separation voltage and detection wavelength of -12 kV and 214 nm were employed， respectively. Furthermore， TCS， TCC， and PCMX showed good linear relationships with their corrected peak areas within a mass concentration range of 1-100 mg/L， and the correlation coefficients （*r*） were all greater than 0.99. Moreover， the limits of detection （LODs， *S/N*=3） and limits of quantification （LOQs， *S/N*=10） were determined to be 0.2 and 1 mg/L， respectively. The spiked recoveries ranged from 94.5% to 104.4% with relative standard deviations of ≤4.8% in all cases. Subsequently， the established method was used to analyze 31 commercial samples， including hand sanitizer， disinfectant， baby powder， and antibacterial cream. A comparative analysis of HPLC， the developed NACE method， and our previously reported micellar electrokinetic chromatographic （MEKC） method was also carried out for the quantitative determination of TCS， TCC， and PCMX. Although no statistically significant differences were observed among the three methods， the results determined for 16 out of the 31 samples did not match the claimed contents. These results therefore indicate the necessity to further control the compositions of disinfectant products. Our results indicate that the newly established NACE method can be an important alternative to HPLC for routine laboratory analyses， especially considering that it minimizes waste generation， requires only a simple sample pretreatment process， and exhibits a good selectivity to the target compounds. It is therefore hoped that the NACE method will be incorporated into the National Standard method in the near future.

随着社会的发展，人们利用消毒剂和抗/抑菌剂预防传染病的意识越来越强，该类产品的产量及种类迅速增多，消毒和抗/抑菌效果主要通过添加抗/抑菌类消毒有效成分实现^[1]^。我国消毒剂原料有效成分清单^[2]^列举的85种成分中，对氯间二甲苯酚（*p*-chloro-*m*-xylenol， PCMX）、三氯生（triclosan， TCS）和三氯卡班（triclocarban， TCC）这3种成分（结构式见图1）作为高效、广谱的消毒有效成分被允许应用于消毒产品中，同时也允许用于个人护理用品如化妆品^[3]^中，TCS还可用于药膏^[4]^中。清单^[2]^中还推荐了每种有效成分的使用范围。化妆品中TCS和TCC的最大允许使用量分别为0.3%和0.2%^[3]^。国标^[5]^还规定消毒产品标签中有效成分应标注其含量范围，并分别制定了这3种有效成分测定的国家标准方法^[6,7]^，以方便监督抽检和消毒效果评价。

**图1:**
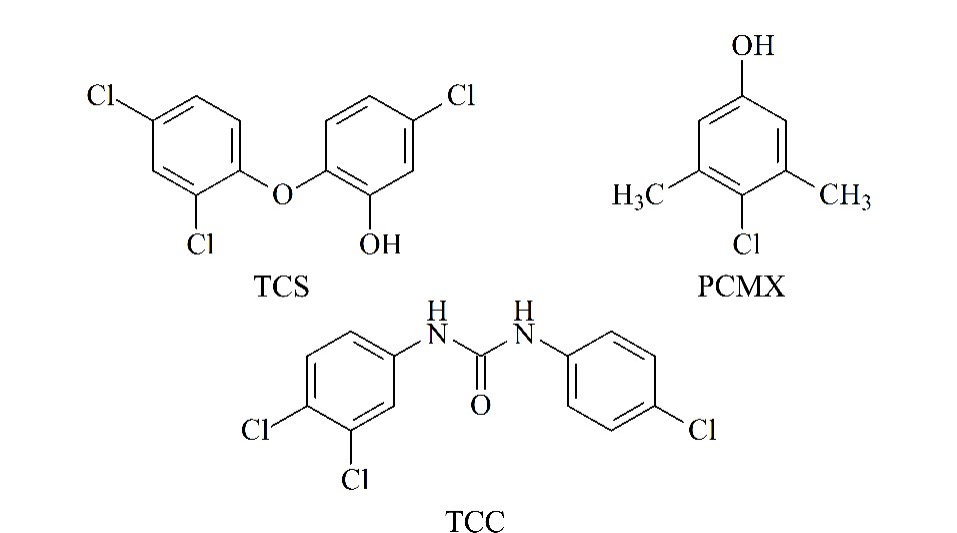
三氯生、对氯间二甲苯酚、三氯卡班的结构式

近年来，我国已经把消毒产品评审注册改革为安全评价备案，产品质量的保证主要依靠市场监管^[8]^，而有效成分的含量检测是常用的监督手段之一。但部分消毒产品的标识含量及检测含量均超过规定技术标准的现象时有发生^[9]^。按目前国标方法对上述3种消毒有效成分进行检测时，经常出现测定结果与标识含量不符的现象，故迫切需要应用毛细管电泳建立与国标方法原理完全不同的新方法，从而确证国标方法的结果。此外，国标方法需使用3种色谱条件且消耗大量对操作者和环境不友好的有机试剂，因此有必要建立一种色谱条件下能同时分析这3种消毒有效成分，且有机试剂消耗更少的分析方法。

本实验室曾以胶束电动毛细管色谱（MEKC）模式实现了PCMX和TCS的同时分析^[10]^。有研究表明：对于溶解性不好的目标物，与其使用大量的表面活性剂，不如使用非水毛细管电泳法（nonaqueous capillary electrophoresis， NACE），可能会获得类似甚至更好的分离效果^[11]^。鉴于TCC不溶于水，若实现上述3种消毒有效成分的同时分析，只能考虑使用非水毛细管电泳模式^[12]^。NACE分析抗抑郁药物^[13]^和手性药物^[14]^已有报道。Buglione等^[15]^研究了电解质及有机溶剂对NACE-非接触式电导检测消毒有效成分季铵盐的影响，但未用于实际样品的测定。Ma等^[16]^建立了NACE分析个人护理品中TCS的方法，但未见个人护理品及消毒产品中TCS、TCC、PCMX同时测定的国内外文献报道。尽管还未见将以上3种消毒有效成分复配在一起的个人护理品、消毒产品及药膏，但已有产品标识其同时含TCS和PCMX，故本文建立了同时分析以上3种消毒有效成分的NACE新方法，并将该研究所建新方法与国家标准方法中的高效液相色谱法（HPLC）以及本实验室以前建立的MEKC法相比较。NACE法极大地减少了有机溶剂的使用，能够准确测定31件样品中上述3种消毒有效成分，且样品前处理简单，非常适合常规实验室分析。

## 1 实验部分

### 1.1 仪器、试剂与材料

PA 800 plus型毛细管电泳仪（配有二极管阵列检测器，美国Beckman Coulter公司）； Waters 2695-2996型液相色谱仪（配有二极管阵列检测器，美国Waters公司）；内径50 μm未涂层熔融石英毛细管（河北永年瑞沣色谱器件有限公司）； Milli-Elix/RiOs型超纯水器（美国Millipore公司）； Vortex-Genie 2涡旋混合器（美国Scientific Industries公司）； SB25-12DTD超声波清洗机（宁波新芝生物科技股份有限公司）； Mettler Toledo XPE 105电子天平（分度值：1 mg，瑞士梅特勒-托利多公司）。Kromasil 100-5-C_18_色谱柱（250 mm×4.6 mm， 5 μm，瑞典AkzoNobel公司）。

氢氧化钠（优级纯）购自北京化工厂；十水合四硼酸钠（分析纯）购自国药集团化学试剂有限公司；聚乙二醇8000（PEG 8000）和聚乙二醇10000（PEG 10000）均购自上海化学试剂总厂；十二烷基三甲基溴化铵（DTAB，纯度>99.0%）、十四烷基三甲基氯化铵（TTAC，纯度>99.0%）和十六烷基三甲基溴化铵（CTAB，纯度>99.0%）均购自北京百灵威科技有限公司。三氯卡班（纯度>98.0%）购自上海阿拉丁公司；甲醇（色谱纯）、乙腈（色谱纯）、聚乙二醇20000（PEG 20000）、三氯生（纯度>97.0%）和对氯间二甲苯酚（纯度>99.0%）均购自美国Sigma-Aldrich公司。

样品：共31件，其中1~10号标示含TCS（7种消毒洗手液、1种消毒凝胶、1种抑菌乳膏和1种药膏）； 11~18号标示含TCC（1种消毒洗手液、1种婴儿皂、2种婴儿爽身粉和4种乳霜）； 19~31号标示含PCMX（5种消毒洗手液、8种消毒液）； 20号样品同时含PCMX和TCS两种成分。消毒剂空白样品由生产厂家提供，其余样品均购于淘宝和京东电商平台。

### 1.2 实验条件

#### 1.2.1 溶液配制

TCS、TCC和PCMX标准储备液的配制：分别准确称取TCS 100.0 mg、TCC 40.0 mg、PCMX 100.0 mg，分别置于10 mL容量瓶中，加入甲醇溶解、稀释、定容，涡旋混匀，制得质量浓度分别为10 g/L TCS、4 g/L TCC和10 g/L PCMX的标准储备液。

TCS、TCC、PCMX混合标准溶液的配制：分别准确吸取3种标准储备液，用样品介质（含5 g/L聚乙二醇20000的甲醇-乙腈（50∶50， v/v））稀释得到质量浓度均为1 g/L的3种混合标准溶液。

TCS、TCC、PCMX混合标准系列工作液的配制：分别准确吸取适量3种标准储备液，用样品介质稀释得到标准工作液，质量浓度分别为：1、10、20、40、60、80和100 mg/L。

#### 1.2.2 样品前处理

非固体样品：一般吸取不少于0.2 mL液体样品或称量不少于0.2 g凝胶样品，置于10 mL塑料离心管中，用样品介质稀释5或10倍，涡旋混匀，超声20 min，冷却后，根据实际样品中目标物的标示含量，稀释至约20 mg/L，进样。

固体样品：婴儿皂样品弃去表层（0.2 cm厚），由洁净手术刀片刮成碎屑进行称量；痱子粉、爽身粉和其他膏状样品：直接称取不少于0.2 g样品，置于10 mL塑料离心管中，用样品介质稀释5或10倍，涡旋混匀（对于难溶膏体可在离心管中加入2颗直径约5 mm干净玻璃珠辅助混匀溶解），超声20 min，冷却后，根据实际样品中目标物的标示含量，稀释至约20 mg/L，进样。

#### 1.2.3 非水毛细管电泳条件

未涂层熔融石英毛细管（50.0 μm×20 cm，总长度30.2 cm）；分离电压：-12 kV；检测波长：214 nm；进样时间：4 s；进样压力：3.448 kPa；分离温度：25 ℃；分离缓冲溶液：含14 mmol/L硼砂、2 g/L PEG 20000和0.5 mmol/L DTAB的甲醇溶液。

新装毛细管分别用1 mol/L NaOH冲洗60 min，超纯水冲洗2 min，分离缓冲溶液冲洗5 min，两次运行之间每次进样前仅用分离缓冲溶液冲洗5 min，保证校正峰面积和迁移时间的重复性。

## 2 结果与讨论

### 2.1 检测波长的选择

用毛细管电泳仪配备的二极管阵列检测器对TCS、TCC和PCMX 3种消毒有效成分进行扫描，TCS在198 nm和280 nm处获得最大吸收峰；TCC在200 nm和264 nm处获得最大吸收峰；PCMX在220 nm和280 nm处获得最大吸收峰。3种消毒有效成分在214 nm均可获得较高灵敏度（见图2），为实现3者的同时测定，尽可能获得高灵敏度同时避免基质干扰，因此选择214 nm为最佳检测波长。

**图2:**
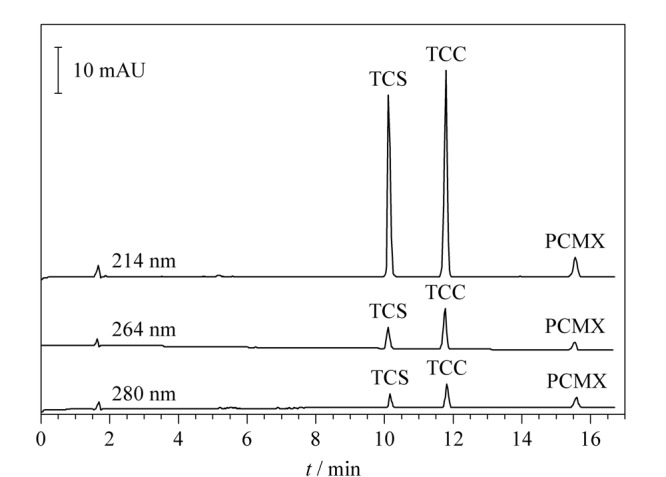
检测波长对3种消毒有效成分检测灵敏度的影响

### 2.2 分离缓冲液及其浓度的选择

#### 2.2.1 分离缓冲溶液中有机溶剂及盐种类的选择

NACE最常用的有机溶剂为甲醇、乙腈或两者的混合溶液^[17]^。甲醇因与弱酸盐如乙酸盐和硼酸盐兼容性好，而成为本研究的首选。硼酸在水溶液中的p*K*_a_为9.24^[18]^，在甲醇中一般会增加2~3个p*K*_a_单位，使TCS和PCMX中的羟基解离而带负电，故本研究选用硼砂，并对其浓度进行优化。

NACE可增加分离的选择性^[19]^，所选用纯甲醇的电导率仅为1.3 μS/cm^[20]^，需加入无机盐增加其导电性。保持分离缓冲溶液中2 g/L PEG 20000和0.5 mmol/L DTAB的含量不变，研究了6、8、10、12、14及16 mmol/L硼砂对混合标准溶液中3种消毒有效成分分离的影响（如图3所示）。随着硼砂浓度的增加，TCS与TCC的分离度逐渐减小，TCC与PCMX的分离度逐渐增加，PCMX与其后系统峰的分离度逐渐增加，迁移时间逐渐缩短，当硼砂浓度大于14 mmol/L时，工作电流增加，焦耳热也相应增加，毛细管内的甲醇溶液沸点低（65 ℃）而易沸腾，导致工作电流中断，无法运行分离程序。此外，各峰分离度基本不再增加。为保证分离度且分离时间尽量短，灵敏度较高且较稳定，优化得到最佳硼砂浓度为14 mmol/L。

**图3:**
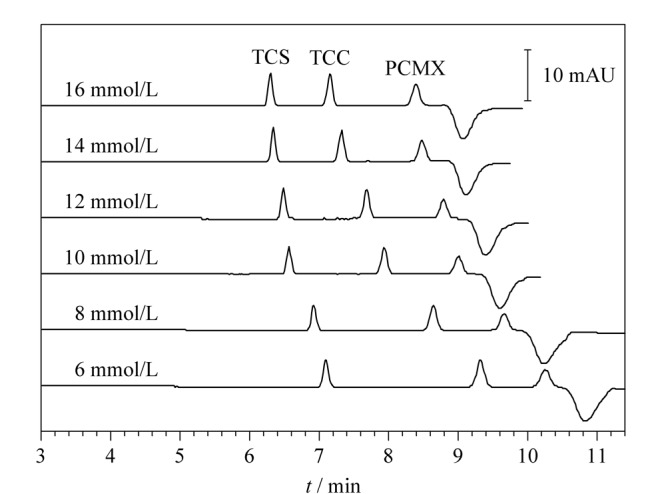
硼砂浓度对3种消毒有效成分分离的影响

#### 2.2.2 分离缓冲溶液中电渗流反转剂及浓度的选择

实验初期，参考文献^[16]^的分离条件，以含14 mmol/L硼砂的甲醇为分离缓冲溶液，甲醇的解离常数p*K*_auto_为16.91^[21]^，甲醇与硼砂的联合作用，使得毛细管内壁带负电，导致TCS和PCMX的酚羟基解离也带负电。对未涂层石英毛细管，当分离缓冲溶液pH>6时，电渗流（electro-osmotic flow， EOF）大于目标物的淌度，故无论待分析物是否带电，均能迁移到检测窗口而被检测^[22]^。然而带负电的TCS和PCMX与EOF的运动方向相反，无法在合理的30 min内迁移到检测窗口。当施加正向电压进样分离时，未观察到任何峰，将电极反向进行加压分离，则出现3个峰，出峰顺序为TCS、TCC、PCMX。然而该条件出峰的重复性并不理想。

0.5 mmol/L CTAB足以使EOF反转且得到稳定EOF^[23]^，故往仅含14 mmol/L硼砂和2 g/L PEG 20000的甲醇分离缓冲溶液中加入0.5 mmol/L CTAB，出峰的重复性得到极大改善，NACE中CTAB的铵阳离子依然优先吸附到管壁上，导致EOF发生反转并得到稳定EOF，与文献^[24]^报道相一致。从CTAB的实验效果得到启发，保持14 mmol/L硼砂和2 g/L PEG 20000不变，尝试并考察了使用其他两种表面活性剂TTAC和DTAB并与CTAB实验效果进行比较，分别研究了浓度均为0.5 mmol/L的CTAB、TTAC和DTAB对混合标准溶液中3种消毒剂有效成分分离的影响（如图4所示），3种表面活性剂烷基链长为CTAB>TTAC>DTAB，随烷基链长的减小，3种消毒剂有效成分的灵敏度、分离度和迁移时间皆有所变化，使用DTAB作为EOF反转剂时虽然灵敏度略低，但分离度尚可，迁移时间最短，足以满足定量要求，本研究最终选择DTAB作为最佳EOF反转剂。

**图4:**
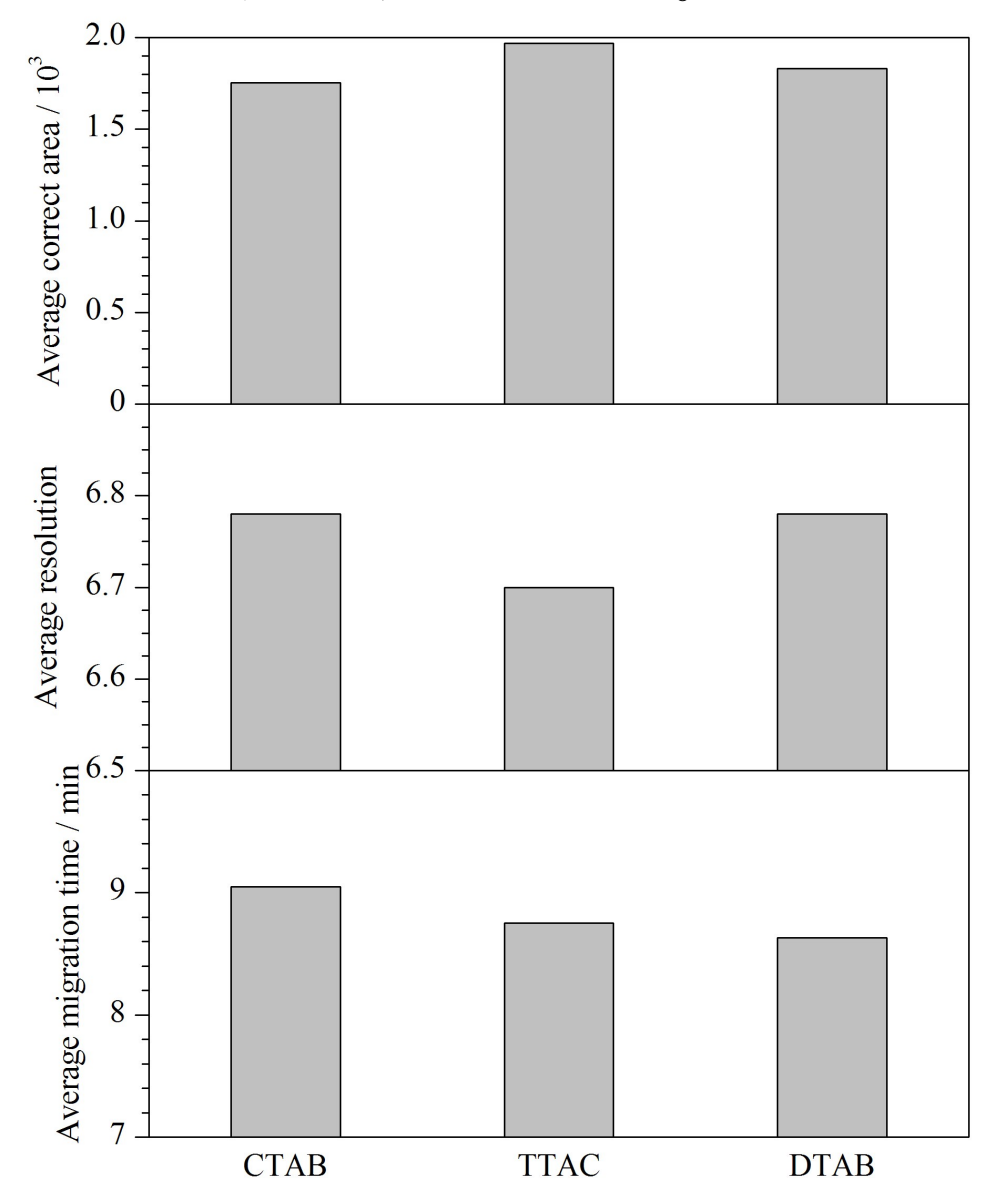
电渗流反转剂对3种消毒有效成分的影响

保持14 mmol/L硼砂和2 g/L PEG 20000不变，分别研究了0.3、0.5和0.7 mmol/L DTAB对混合标准溶液中3种消毒有效成分分离的影响（见图5）：当DTAB浓度为0.5 mmol/L时灵敏度最高，分离度最大，迁移时间最短。综合考虑，DTAB最佳浓度为0.5 mmol/L。

**图5:**
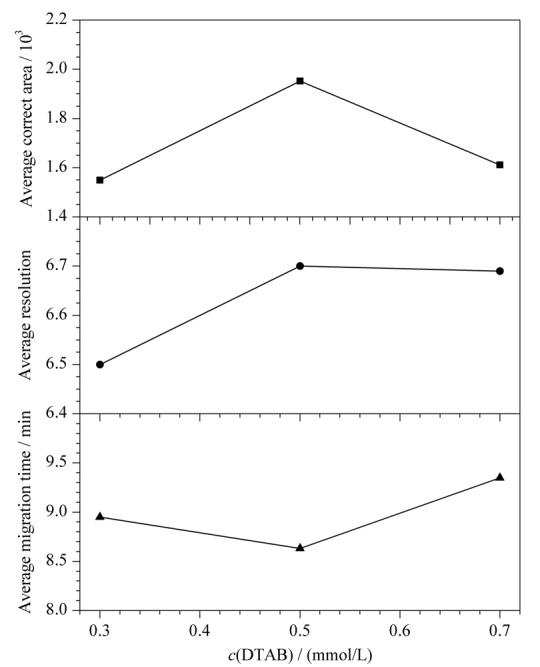
DTAB浓度对3种消毒有效成分分离的影响

#### 2.2.3 分离缓冲溶液中PEG相对分子质量及浓度的选择

TCS和PCMX为酚类物质，极易吸附在毛细管内壁，PEG类高分子聚合物已被证明具有良好的亲水性和抑制吸附的能力^[25]^。当分离缓冲溶液中仅含14 mmol/L硼砂和0.5 mmol/L DTAB的甲醇溶液时，各峰迁移时间的重复性不好。有文献报道PEG 20000涂层的毛细管可提高NACE中迁移时间的重复性^[26]^。本研究将PEG 20000作为动态涂层，以期获得与涂层管一样的抑制吸附的效果。保持14 mmol/L硼砂和0.5 mmol/L DTAB不变，分别研究了2 g/L相对分子质量分别为8000、10000和20000的PEG对混合标准溶液中3种消毒有效成分分离的影响（如图6所示）：随着PEG相对分子质量的增加，灵敏度和分离度先逐渐减少，然后又逐渐增加，迁移时间逐渐减少。综合考虑，最终选择PEG 20000作为吸附抑制剂。

**图6:**
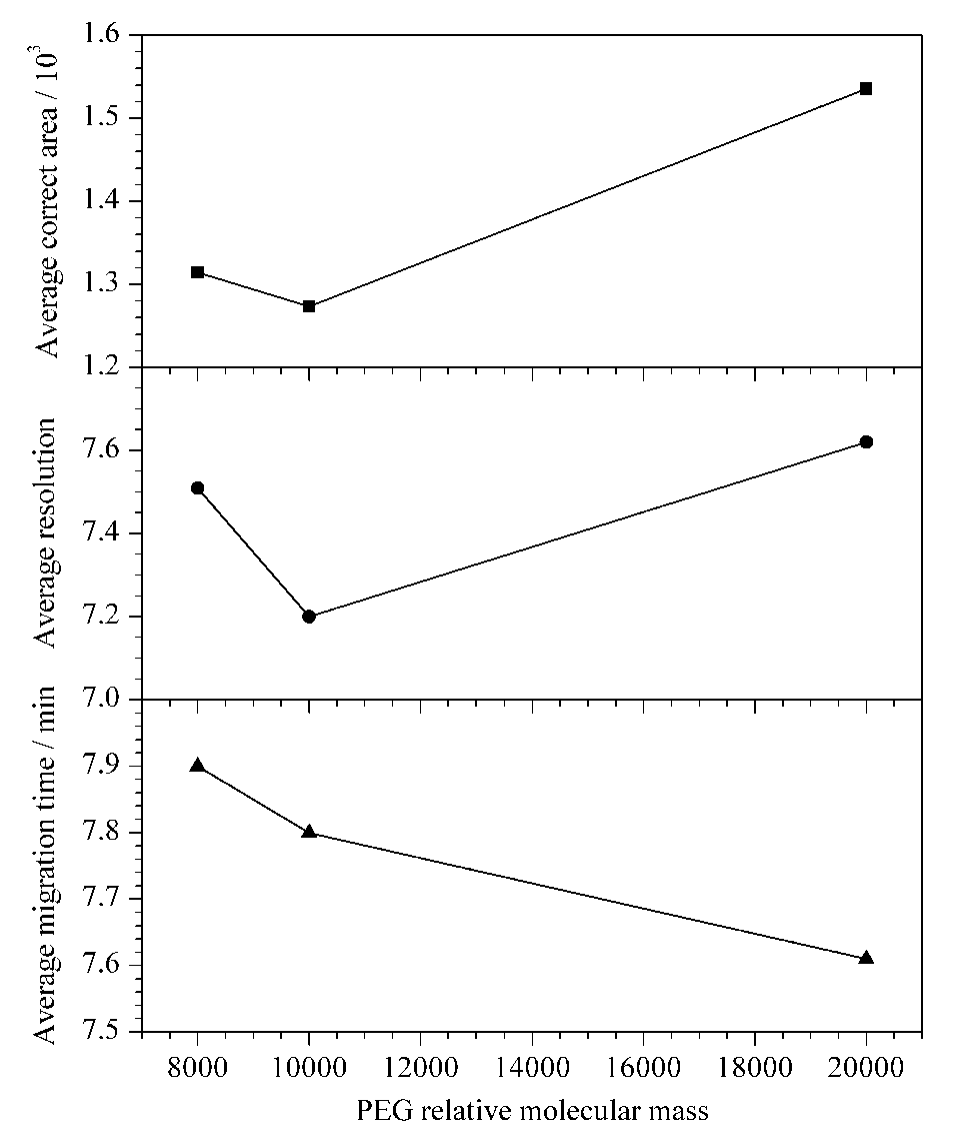
PEG相对分子质量对3种消毒有效成分混合标准溶液分离的影响

保持14 mmol/L硼砂和0.5 mmol/L DTAB不变，分别研究了PEG 20000不同质量浓度（2、5和8 g/L）对混合标准溶液中3种消毒有效成分分离的影响（见图7）：随着PEG 20000质量浓度的增加，灵敏度下降，分离度逐渐减少，迁移时间逐渐增加。综合考虑，2 g/L PEG 20000为最佳选择。

**图7:**
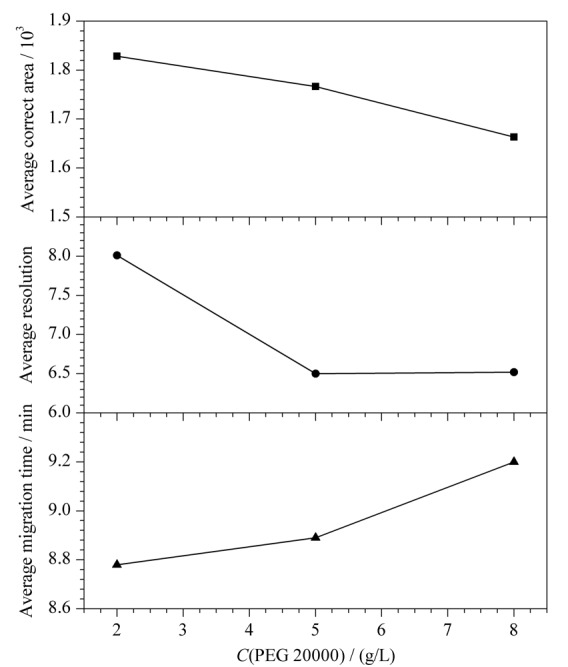
PEG 20000质量浓度对3种消毒有效成分分离的影响

### 2.3 分离电压的选择

本实验所用分离缓冲液中硼砂最佳浓度为14 mmol/L，限制了过高电压的使用。保持石英毛细管长和内径不变，比较了-8、-10、-12、-14和-16 kV分离电压对混合标准溶液中3种消毒有效成分分离的影响：随着分离电压的增加，迁移时间缩短、峰高增加，但过高的电压使工作电流增加，产生过多的焦耳热，易造成工作电流中断。当电压为-12 kV时，电流仅为12 μA，且较为稳定，结合灵敏度、分离度、重复性考虑，-12 kV为最佳选择。

### 2.4 样品介质的选择

样品介质在准确定量中发挥极为重要的作用：合适的样品介质既可增加分离及定量的重复性，还可增加样品的溶解度和检测灵敏度。鉴于TCC不溶于水，欲实现上述3种消毒有效成分的同时分离，必须解决TCC的溶解性问题。

#### 2.4.1 样品介质中有机溶剂的选择

TCS与TCC的正辛醇/水分配系数（log *K*_ow_）分别为4.8和4.9，属于亲脂性较强的化合物^[27]^。TCC不溶于水，但溶于烷醇酰胺、脂肪醇聚氧乙烯醚等非离子表面活性剂^[16]^。但这些溶剂均为不常用有机溶剂，故实验初期首选常用的甲醇、乙醇和乙腈溶解这3种消毒有效成分。PCMX和TCS均易溶解在这3种常用有机溶剂中，可配制成10 g/L储备液，室温放置无析出。TCC在甲醇中的溶解性最好，可配制成4 g/L储备液，室温放置无析出，将4 g/L TCC通过稀释制备成100 mg/L或10 mg/L标准工作液时，只要样品介质中含有水，则导致TCC析出，欲实现3种消毒有效成分的同时分离，标准工作液必须用纯有机溶剂稀释。

有机溶剂组成是影响迁移时间、灵敏度和峰形的关键因素之一，当使用分离缓冲液作为样品介质时，样品介质与分离缓冲液之间无电导差异，导致峰展宽^[16]^，不利于准确定量。往样品介质中添加一定比例的乙腈，测试了4个不同体积比的甲醇和乙腈（80∶20、60∶40、50∶50和40∶60）对3种消毒有效成分分离的影响：当100%甲醇时，3种消毒有效成分峰形和灵敏度均不佳，使用100%乙腈时，峰形虽有所改善，但灵敏度较低；80∶20的比例易导致造成电流异常或中断；60∶40的比例则导致各峰灵敏度降低；40∶60比例使3种消毒有效成分无法完全分离，仅得到TCS和TCC这两种有效成分的峰；50∶50比例时，则3种消毒有效成分峰形和灵敏度好，迁移时间短。故甲醇与乙腈的最佳体积比为50∶50。

#### 2.4.2 样品介质中PEG 20000含量的优化

受分离缓冲液中添加PEG 20000以抑制3种消毒有效成分在管壁吸附的启发，在样品介质中添加PEG 20000也将使3种消毒有效成分在管内壁的吸附降低，各峰灵敏度增加，实验条件更加稳定，定量重复性更好。研究了0、2、5及10 g/L PEG 20000对混合标准溶液中3种消毒有效成分分离的影响（如图8所示）：当PEG 20000质量浓度为5 g/L时，灵敏度最高，分离度尚可，迁移时间较短，综合考虑，5 g/L PEG 20000为最佳选择。

**图8:**
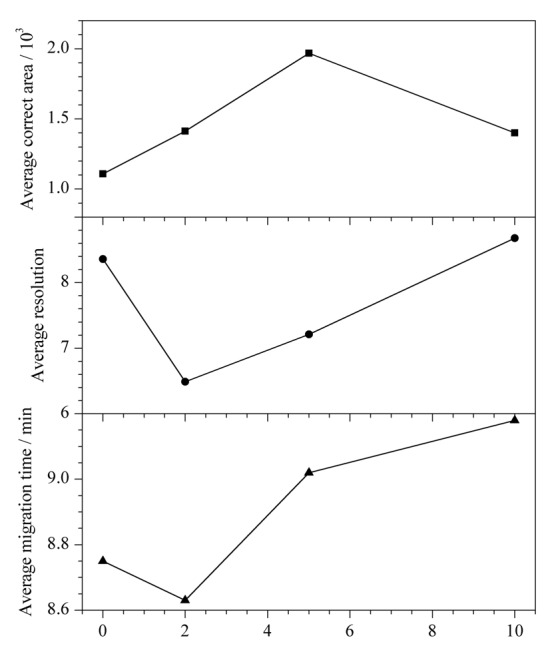
样品介质中PEG 20000质量浓度对3种消毒有效成分分离的影响

综上，经过对各关键因素的优化，得到最佳电泳条件，在此最佳条件下对混合标准溶液中3种消毒有效成分进行分离（见图9）：按《美国药典》方法计算各峰的理论塔板数（单次计算）分别为55431、42145、34516；分离度（单次计算）分别为10.323、7.342，远高于基线分离时的1.5的分离度，故该法可以对实际样品进行分析。

**图9:**
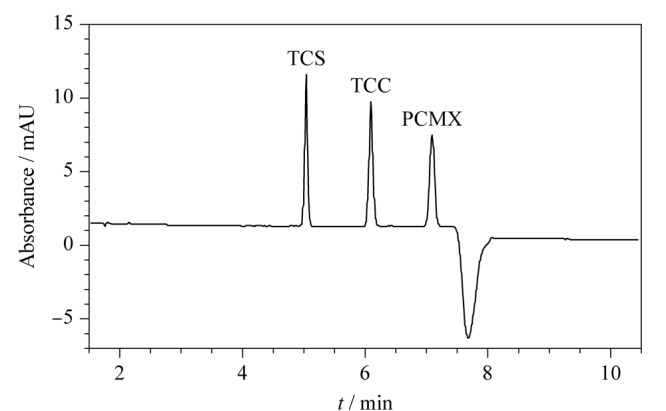
最佳电泳条件下3种消毒有效成分混合标准溶液的电泳图

### 2.5 方法学考察

#### 2.5.1 标准曲线及检出限

在上述最佳电泳条件下，将TCS、TCC和PCMX的混合标准储备液用样品介质逐级稀释，配制成混合标准溶液，质量浓度分别为1、10、20、40、60、80和100 mg/L，依次进样分析，外标法定量，校正峰面积（*Y*，峰面积除以迁移时间）与质量浓度（*X*， mg/L）具有良好线性关系。线性回归方程、线性范围、相关系数（*r*）、检出限（LOD，信噪比为3）及定量限（LOQ，信噪比为10）见表1。

**表1:** 3种消毒有效成分的回归方程、线性范围、相关系数、检出限及定量限

Compound	Regression equation	Linear range/ （mg/L）	*r*	LOD/ （mg/L）	LOQ/ （mg/L）
TCS	*Y*=126.01*X*+175.61	1-100	0.9991	0.2	1.0
TCC	*Y*=123.55*X*+178.31	1-100	0.9983	0.2	1.0
PCMX	*Y*=108.39*X*+121.93	1-100	0.9984	0.2	1.0

*Y*：corrected peak area；*X*：mass concentration，mg/L.

#### 2.5.2 仪器精密度

分别在4.00、40.0和80.0 mg/L 3个水平下进行仪器精密度试验，将配制好的低、中、高3个水平的标准溶液在最佳NACE条件下分别连续进样7次进行测定，计算迁移时间和校正峰面积的相对标准偏差（RSD），迁移时间重复性小于1.5%，校正峰面积重复性小于5%。

#### 2.5.3 方法精密度

取20 mL仅含有PCMX的实际样品中分别加入60 mg TCS和100 mg TCC，制成均含3种消毒有效成分的样品，进行方法精密度实验，考察方法的适用性。平行制备7份样品，根据1.2.2节进行样品前处理。在最佳NACE条件下进样测定，计算测定3种消毒有效成分含量的RSD，考察方法的日内精密度；按照上述方法对此自制样品连续测定7天，每日平行测定3次，计算测定3种消毒有效成分含量的RSD，考察方法的日间精密度，TCS、TCC、PCMX含量的日内精密度分别为1.7%、1.3%和2.3%，日间精密度分别为3.3%、4.0%和2.2%。

#### 2.5.4 加标回收率

对空白消毒剂样品进行加标回收试验，加标水平为4.00、40.0和80.0 mg/L，每个加标水平平行处理7份，3种消毒有效成分的加标回收率见表2。

**表2:** 3种消毒有效成分在低、中、高3个水平下的加标回收率（*n*=7）

Analyte	Added/ （mg/L）	Found/ （mg/L）	Recovery/ %	RSD/ %
TCS	4.00	4.01	100.3	4.4
	40.0	37.9	94.7	4.8
	80.0	83.5	104.3	1.7
TCC	4.00	3.92	97.9	4.3
	40.0	38.4	96.0	3.8
	80.0	82.9	103.6	1.3
PCMX	4.00	4.13	103.3	4.6
	40.0	38.0	94.5	3.2
	80.0	83.6	104.4	2.3

### 2.6 实际样品测定

按1.2.2节所述样品前处理方法，对1~31号消毒样品进行NACE测定，每件样品平行处理3份。用本实验室之前建立的MEKC法^[10]^对含TCS、PCMX的23件样品进行测定，并分别参照国标方法GB/T 27947-2020和GB/T 34856-2017对1~31号样品中的PCMX和TCS^[6]^、TCC^[7]^进行测定，并进行了部分条件优化。3种消毒有效成分的分离测定均使用同一根色谱柱。流动相均为甲醇-水，比例分别为70∶30、80∶20和75∶25（v/v）。流速均为1.0 mL/min；进样量均为10 μL；柱温均为30 ℃。PCMX及TCS的检测波长均为280 nm； TCC检测波长为264 nm。3种方法的测定结果见表3，应用统计学软件SPSS 25对TCS和PCMX的3种方法、TCC两种方法的结果进行单因素方差分析，*P*分别为0.817和0.974， 3种或2种方法的测定结果无统计学显著性差异，证明了所建NACE方法的准确性。其中1号样品因存在较明显的基质干扰，无法用MEKC进行准确定量，而NACE对31件样品分析中均未见干扰，证明NACE的选择性高于HPLC和MEKC，利于得到准确的定量结果。

**表3:** 实际样品中3种消毒有效成分的测定结果

Sample No.	Analyte	Specified content/%	Determined contents/%		
			NACE	HPLC^a^	MEKC^b^
1	TCS	0.045-0.055	0.052	0.052	-
2		0.045-0.055	0.053	0.049	0.054
3		0.045-0.065	0.031	0.031	0.05
4		0.20-0.24	0.21	0.18	0.22
5		0.12-0.14	0.11	0.11	0.13
6		0.25-0.30	0.25	0.25	0.27
7		0.09-0.11	0.08	0.08	0.08
8		0.50-0.60	0.53	0.53	0.54
9		0.30	0.14	0.15	0.22
10		1.00	0.75	0.87	1.13
11	TCC	0.05-0.06	0.04	0.04	/
12		yn	0.14	0.15	/
13		yn	0.09	0.10	/
14		yn	0.07	0.08	/
15		yn	0.11	0.13	/
16		yn	0.72	0.72	/
17		yn	0.13	0.12	/
18		yn	0.05	0.04	/
19	PCMX	0.18-0.22	0.19	0.20	0.14
20		0.09-0.11	0.08	0.06	0.08
21		0.18-0.22	0.23	0.20	0.15
22		0.52-0.64	0.71	0.42	0.46
23		0.27-0.33	0.22	0.25	0.15
24		0.25-0.45	0.13	0.15	0.13
25		4.30-5.30	3.70	4.90	3.70
26		4.50-5.50	5.50	5.50	4.40
27		4.50-5.50	1.06	1.16	1.10
28		2.5	1.88	1.80	1.80
29		2.30-2.80	0.23	0.24	0.10
30		2.50-3.00	1.53	2.03	1.80
31		2.30-2.80	1.28	1.97	1.50

-：Content cannot be quantified due to interferences. yn：Content was not specified. /：Content could not be analyzed by MEKC due to its insolubility in water. a：TCS and PCMX were analyzed according to GB/T 27947-2020，and TCC was analyzed according to GB/T 34856-2017. b：Compounds were analyzed according to reference^[10]^.

## 3 结论

本研究建立了消毒剂、个人护理品及药膏中TCS、TCC和PCMX同时分析的NACE新方法，对分离缓冲溶液及样品介质进行了充分优化。该法选择性好，样品前处理简单，仅需稀释即可直接进样，得到的电泳图无干扰。与国标方法相比，极大地减少了有机溶剂的消耗和检测成本，是国标方法的有益补充，希望将来能纳入国标方法，发挥更大作用。此外，检测结果与标识含量不符的情况依然存在，给消毒安全带来隐患，需加强消毒产品的监管。
